# Ti–O–Cu Nanotubular Mixed Oxide Grown on a TiCu Alloy as an Efficient Material for Simultaneous Photoelectrocatalytic Oxidation and PMS Activation for Pollutant Degradation

**DOI:** 10.1021/acsomega.4c07301

**Published:** 2024-11-13

**Authors:** Carolyne I. P. Crivelli, Juliana de Almeida, Cleber A. Lindino, Lucio C. de Almeida, Christiane A. Rodrigues, Guilherme G. Bessegato

**Affiliations:** † Universidade Estadual do Oeste do Paraná (UNIOESTE), Toledo Campus, Rua da Faculdade 645, 85903-000 Toledo, Parana, Brazil; ‡ Universidade Federal de São Paulo (UNIFESP), Rua São Nicolau 210, 09913-030 Diadema, São Paulo, Brazil; § Department of Chemistry, Universidade Estadual de Londrina (UEL), Rodovia Celso Garcia Cid, PR 445 km 380, 86057-970 Londrina, Parana, Brazil; ∥ Universidade Tecnológica Federal do Paraná (UTFPR), Dois Vizinhos Campus, Estrada para Boa Esperança, km 04, 85660-000 Dois Vizinhos, Parana, Brazil

## Abstract

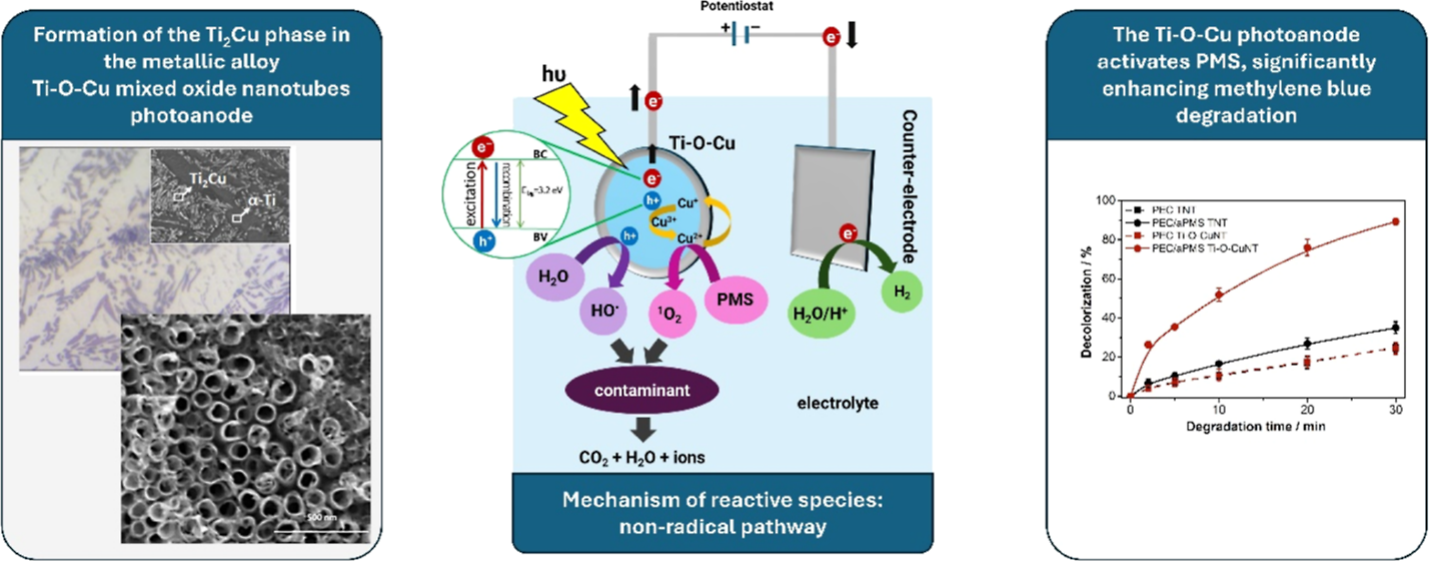

This study introduces a novel photoelectrocatalytic (PEC) system featuring a Ti–O–Cu mixed nanotubular oxide photoanode for the simultaneous activation of peroxymonosulfate (PMS), targeting the removal of emerging contaminants, such as methylene blue dye, tetracycline, and ibuprofen. The Ti–5.5Cu (atom %) alloy substrate and the nanotubular oxide layer were synthesized through arc melting and electrochemical anodization. The conditions of photoelectrocatalysis-assisted PMS activation (PEC/aPMS) were optimized using experimental design, achieving 90.4% decolorization of methylene blue dye within 30 min under optimal conditions: pH 4, an applied potential of 0.5 V vs Ag/AgCl, and a PMS concentration 50 times the molar concentration of the contaminant, utilizing a 10 W UV LED at 365 nm. In contrast, only 25% decolorization was observed without PMS. Singlet oxygen (^1^O_2_) was identified as the primary pathway for PMS activation (nonradical). Additionally, the PEC/aPMS system effectively degraded model contaminants, achieving 52% degradation of ibuprofen, 78% of methylene blue, and 92% of tetracycline in 10 mg L^–1^ total organic carbon solutions within 60 min under optimized conditions. The electrode exhibited remarkable stability, maintaining its efficiency throughout the experiments. These findings highlight the potential of mixed nanostructured oxide electrodes for developing highly efficient and durable PEC systems with integrated PMS activation for the removal of organic contaminants.

## 1 Introduction

The contamination of water sources has become a pressing environmental issue due to the toxicity and persistence of pollutants released into the environment. Approximately 80,000 chemical compounds, totaling 500 million tons, are discharged annually, many categorized as contaminants of emerging concern (CECs). These include pharmaceuticals, pesticides, personal care products, flame retardants, and perfluoroalkyl substances, among others.^1^ CECs can act as endocrine disruptors, posing significant health risks to humans and wildlife.^1,2^ Despite the severity of this issue, current water treatment plants often fail to adequately monitor or regulate these contaminants, presenting a challenge in their detection and removal from the environment. Conventional wastewater treatment plants, while crucial, struggle to remove and degrade many CECs effectively,^2−4^ highlighting the need for advanced solutions.

Advanced oxidation processes (AOPs) are methods used to remove contaminants from water sources by generating highly reactive species such as hydroxyl radicals (^•^OH) or other reactive species.^3,4^ These species can effectively break down a wide range of contaminants, resulting in byproducts with decreased toxicity.^5,6^ In recent years, peroxymonosulfate (PMS) activation has garnered attention as a viable alternative for contaminant removal. PMS, available commercially as Oxone (2KHSO_5_·KHSO_4_·K_2_SO_4_), is a potent oxidant (*E*^0^ = 1.85 V vs NHE) but exhibits limited reactivity toward organic contaminants.^7^ PMS can be activated through heat, light, or catalysis, with catalytic activation being a more efficient and low-temperature alternative.^8^ A range of catalysts, including transition metals, noble metals, and metal oxides, have been used in the catalytic activation of PMS. These methods help produce sulfate radicals and other reactive species that effectively degrade pollutants.^8−10^

Various heterogeneous catalysts, primarily metal oxides and some carbonaceous (metal-free) materials, have been investigated for PMS activation.^7^ However, most of these catalysts are used in dispersion form, often as nanoparticles, which poses significant challenges for recovery and reuse, requiring additional filtration steps. In contrast, immobilized catalysts offer a distinct advantage as they can be more easily recovered and reused without complex separation processes. This highlights the potential of immobilized catalysts, which offer scalability, enhanced stability, and efficient pollutant degradation. Notably, recent studies have explored the activation of PMS within a photoelectrocatalysis (PEC) system.^11−16^ A photoelectrocatalytic oxidation process typically involves an *n*-type photoelectrode that generates electrons and holes upon light absorption and a cathode that receives electrons directed by the electrochemical potential difference. These e^–^/h^+^ pairs can also participate in redox reactions with PMS. While previous research predominantly utilized catalyst cathodes to activate PMS, such as platinum,^11^ CuO_2_,^14^ and polydopamine-modified carbon felt cathodes,^13^ there is growing interest in the potential of photoanodes to achieve PMS activation. The photoelectrode can be made from various materials, including semiconductors and metal oxides, and the choice of material depends on the desired properties, such as bandgap, light absorption, and electron transfer. For example, Bacha et al.^16^ developed a Co-BiVO_4_/FTO photoanode that showed significant PMS activation compared to other photocatalysts. Zheng and colleagues prepared a modified molybdenum disulfide-embedded carbon cloth photoanode for PMS activation.^15^ However, the development of efficient photoanodes for this purpose remains in its nascent stages, necessitating further research to optimize the performance and elucidate mechanisms of PMS activation.

Inspired by these challenges, in this work, we report the synthesis of a novel Ti–O–Cu mixed nanotubular oxide photoanode directly grown on a TiCu alloy through a simple and controllable electrochemical anodization process. This unique material combines the benefits of copper doping and a nanotubular structure to enhance light absorption, promote charge separation, and improve catalytic activity for pollutant degradation. This is the first time a Ti–O–Cu mixed nanotubular oxide photoanode has been synthesized and applied for simultaneous photoelectrocatalytic degradation and PMS activation to remove emerging contaminants. Given the limited understanding of PMS activation in photoelectrocatalytic systems, developing multifunctional materials such as the Ti–O–Cu mixed nanotubular oxide is crucial for advancing the removal of emerging contaminants through AOPs. The photoanode, comprising a Ti–O–Cu mixed nanotubular oxide prepared through electrochemical anodization of a Ti–Cu alloy, offers a highly stable oxide film with excellent surface area and electron transport properties.^17^

## 2 Experimental Section

### 2.1 Preparation of Ti–Cu Alloys and Characterization

Ti–5.5Cu (atom %) in atomic composition was prepared in an arc furnace under an inert atmosphere using 99.9% titanium (Ti-Brasil) and 99.9% copper (Alfa Aesar) as raw materials. During the preparation of the alloy, the lost mass was lower than 0.02 wt % in relation to the nominal composition. The ingot with 20 g was maintained at 1050 °C for 7200 min for homogenization and then cooled down to 1.0 °C min^–1^ in a vacuum furnace until it reached room temperature. After this, the ingot was chopped into a disk shape (∼2.0 cm diameter and ∼1.0 mm thickness), and the elemental composition was verified using an X-ray fluorescence spectrometer (XRF—BRUKER S8 Tiger). For microstructural analysis, discs were polished and etched with a mixed acid solution (1.5% HCl, 2.5% HNO_3_, and 1.0% HF in water). The morphological and compositional characterization of the sample was carried out using an optical microscope, Zeiss Axiolab 5, connected with a Cam Zeiss AxioCam ERc 5s, and scanning electron microscopy (SEM) JEOL-LV 6600 coupled with energy-dispersive X-ray spectroscopy (EDX). The crystalline phases of the alloys were determined by X-ray diffraction (XRD) using a Bruker D8 Advance AXS diffractometer, Cu Kα radiation, a Bragg–Brentano optical setup, and a Lynxeye detector. The angular range covered was 2θ = 10–90° with a step size of 0.02°. The Rietveld method was applied to XRD data to quantify the substrate phases (using TOPAS software).

### 2.2 Nanotube Array Electrode Preparation

The nanotubular oxide layer was produced by the electrochemical anodization of Ti–5.5Cu alloy discs in a two-electrode cell configuration. The cell included a platinum spiral as the counter electrode and an alloy disc as the working electrode. The electrolyte comprised 0.2 M HF in ethylene glycol with 3.5% (v/v) H_2_O. Anodization was carried out at 30 V for 120 min using a DC power supply. After anodization, the electrodes were rinsed with distilled water to remove residual electrolytes and soaked in ethanol for 24 h to remove the nanograss. Nanograss refers to a type of surface morphology characterized by the presence of very short and thin nanowires on top of the underlying nanotube structure. Finally, the electrode was annealed at 450 °C for 150 min in atmospheric air in a muffle furnace (EDG-F3000) to convert amorphous structures into crystalline phases. This electrode with the layer of a nanotubular mixed oxide of titanium and copper was named Ti–O–CuNT. Similarly, pure TiO_2_ oxide electrodes (TNT) were produced using grade 2 titanium sheets (∼99.7%, Realum, Brazil) under identical conditions.

### 2.3 Physical–Chemical and Photocharacterization

The morphology and elemental composition of Ti–O–CuNT were examined using field emission scanning electron microscopy (SEM-FEG, FEI Inspect F50) and energy-dispersive X-ray spectroscopy (EDX), respectively. High-resolution SEM images were taken at 200,000× with an acceleration voltage of 5.0 kV and a current of 1.0 × 10^–8^ A. Cross-sectional analysis for EDX was prepared via focused ion beam (FEI Helios G4 CX Dual Beam). The chemical states of the elements in the oxide layer were identified using X-ray photoelectron spectroscopy (XPS, K-alpha, Thermo Scientific) with a monochromatic Al Kα X-ray source (*h*ν = 1486.6 eV) operating at 150 W under a pressure fixed to 4.8 × 10^–9^ mbar. The sample was irradiated for one hour in a typical setup with the X-ray source at 45° from the sample surface while the angle between the analyzer and the sample surface was 90°. Spectra were charge-corrected to the main line of the C 1s (aromatic carbon) set to 284.7 eV and deconvoluted by OriginPro software using linear background subtraction and fitted with a Gaussian function. The peak positions were determined with ±0.1 eV accuracy. Crystalline phases were determined using an X-ray diffractometer (XRD) (Bruker D8 ADVANCE) with Cu Kα radiation (λ = 1.5406 Å), a Ni filter, and a parallel beam optical setup. The patterns were obtained at room temperature, and the angular range covered was 2θ = 24–48° with a step size of 0.02°. Diffuse reflectance spectroscopy (DRS) spectra in the ultraviolet–visible region were recorded between 200 and 800 nm at ambient temperature using a spectrophotometer (UV-2600 Shimadzu) with an integrating sphere module for diffuse reflectance.

Photoelectrochemical characteristics of TNT and Ti–O–CuNT were evaluated using current versus potential curves in the presence and absence of irradiation. Linear scanning voltammograms were taken at −0.5 to 1.5 V vs Ag/AgCl (3 M KCl) in a 0.1 M Na_2_SO_4_ electrolyte at a scan rate of 20 mV s^–1^. The semiconductor electrode was irradiated by a 10 W UV LED (365 nm) positioned 5 mm from the external wall of the cell. A 50 mL highly transparent polystyrene cell was used to accommodate the working electrode (TNT or Ti–O–CuNT), counter electrode (stainless steel), and reference electrode (Ag/AgCl/KCl 3 M). An Ivium Vertex potentiostat was used for these measurements.

### 2.4 Activation of PMS in a Photoelectrocatalytic System

The mixed oxide electrode (Ti–O–CuNT) was applied in the photoelectrocatalytic degradation of the methylene blue (MB) dye solution by adding PMS (PEC/aPMS system). To determine the performance of the Ti–O–CuNT electrode in activating the PMS, a 5.0 mg L^–1^ solution (1.56 × 10^–5^ M) of methylene blue (MB) dye was used as a model contaminant containing a 0.1 M Na_2_SO_4_ electrolyte. The initial concentration of PMS in the reactor was 0.468 mM, which refers to 30 times the MB molar concentration (0.1 mL of a PMS stock solution of 0.117 M was added to the cell). The PEC cell was a highly transparent polystyrene flask (50 mL) filled with 25.0 mL of the work solution.

The electrochemical setup featured a photoanode as the working electrode, comprising either Ti–O–CuNT or TNT (0.283 cm^2^), a stainless-steel counter electrode (2.0 × 2.0 cm), and a Ag/AgCl reference electrode immersed in a KCl solution (3 M). The measurements were conducted using an Ivium Vertex potentiostat. A 10 W UV LED, emitting at a maximum wavelength of 365 nm (below TiO_2_’s common band gap of 390 nm), was positioned 5 mm from the external wall of the cell to illuminate the photoanode, situated close to this wall. Continuous agitation was maintained at 500 rpm throughout the experiments, and 2 mL of the solution was periodically collected from the reactor at 2, 5, 10, 20, and 30 min intervals. The collected samples were subsequently assessed for decolorization of the methylene blue (MB) dye using a UV/vis absorption spectrophotometer, specifically at 664 nm (Shimadzu UV 1800-PC). Following each measurement, collected aliquots were reintroduced into the cell. Every degradation experiment was conducted at least in duplicate, ensuring the reliability and consistency of our results.

### 2.5 Experimental Design

A full factorial design (FFED) with three independent variables (pH, applied potential, and PMS concentration) was applied for the optimization of MB degradation solution by the PEC/aPMS process using the response surface methodology^18^ (see Table 1). Assays corresponding to the upper and lower values of the independent variables were conducted in duplicate. In contrast, a central point test was additionally performed in triplicate to evaluate the pure error of the MB degradation experiments. The independent variables, namely, solution pH, applied potential (*E*_app_), and PMS concentration, were explored in the ranges of 4.00–8.00, 0.5–1.5 V (vs Ag/AgCl), and 0.156–0.780 mmol L^–1^, respectively. Subsequently, three levels for each *i*th independent variable (i.e., −1, 0, +1) were chosen and coded according to eq 1
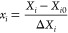
1where *x*_*i*_ is the coded level, *X*_*i*_ is the real value, *X*_*i*0_ is its real value in the central point, and Δ*X*_*i*_ is half of the difference between the upper and lower values of the *i*th independent variables. Table 1 presents the coded and real values for the three independent variables. Decolorization efficiency (DE/%) was chosen as a response and correlated with the coded values of the variables to generate a general first-order polynomial eq 2 by using the least-squares method^18−20^
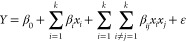
2where *Y* is the observed response (i.e., DE/%), β_0_ is a constant coefficient, β_*i*_ is the linear effect, and β_*ij*_ is the interaction effect between the independent variables, respectively. The terms *k* and ε are the number of independent variables and the random error, respectively. The generated polynomial equation was then used to construct response surfaces (with StatSoft Statistica version 10 software) to determine the optimal conditions for MB degradation. All degradation assays were randomly conducted to minimize systematic errors in the decolorization efficiencies.

**Table 1:** Experimental Design Matrix with Response Values for MB Decolorization by Photoelectrocatalysis with Activated PMS (PEC/aPMS) Using a Ti–O–CuNT Photoanode^a^

run	coded levels			real values^b^			observed response
	*X*_1_	*X*_2_	*X*_3_	*X*_1_^a^	*X*_2_^b^	*X*_3_^c^	DE/%
1	–1	–1	–1	4.00	0.5	0.156	86.6
2	–1	–1	–1	4.00	0.5	0.156	83.3
3	1	–1	–1	8.00	0.5	0.156	28.9
4	1	–1	–1	8.00	0.5	0.156	35.1
5	–1	1	–1	4.00	1.5	0.156	63.9
6	–1	1	–1	4.00	1.5	0.156	57.7
7	1	1	–1	8.00	1.5	0.156	32.8
8	1	1	–1	8.00	1.5	0.156	34.7
9	–1	–1	1	4.00	0.5	0.780	90.4
10	–1	–1	1	4.00	0.5	0.780	88.1
11	1	–1	1	8.00	0.5	0.780	55.9
12	1	–1	1	8.00	0.5	0.780	52.5
13	–1	1	1	4.00	1.5	0.780	80.4
14	–1	1	1	4.00	1.5	0.780	76.2
15	1	1	1	8.00	1.5	0.780	46.3
16	1	1	1	8.00	1.5	0.780	47.4
17	0	0	0	6.00	1.0	0.468	58.1
18	0	0	0	6.00	1.0	0.468	64.2
19	0	0	0	6.00	1.0	0.468	57.7

a Conditions: 25 mL of MB 5 mg L^–1^ in Na_2_SO_4_ 0.1 M; irradiation by a 10 W UV LED at 365 nm.

b Real values: *X*_1_^a^ = pH, *X*_2_^b^ = *E*_app_ (V vs Ag/AgCl), and *X*_3_^c^ = PMS concentration (mmol L^–1^).

In cases involving the addition of PMS, a volume of a 0.04 M PMS stock solution prepared in ultrapure water was introduced into the cell to achieve the final concentrations, as detailed in Table 1.

After the conditions for degrading the MB solution were optimized, experiments were conducted to compare various treatments and assess the contribution of each factor to PMS activation. These treatments included direct oxidation by PMS, PMS + UV radiation, PMS + electrocatalysis, PMS + photocatalysis, photocatalysis alone, photolysis, and pure catalysis (free electrode in the presence of PMS).

### 2.6 Mechanism of Generation of Reactive Species

The contribution of reactive species ^•^OH, SO_4_^•–^, and ^1^O_2_ was investigated through quenching experiments using the appropriate scavengers. Methyl alcohol (MeOH), *tert*-butyl alcohol (TBA), and sodium azide (SA) were employed as quenchers for ^•^OH/SO_4_^•–^, ^•^OH, and ^1^O_2_, respectively.^21,22^ In a typical degradation experiment outlined in Section 2.4, a 50× quantity (molar ratio) of each scavenger was added to the PEC/aPMS system relative to the concentration of PMS in 25 mL of the working solution. The decolorization of the MB solution was monitored by using the same approach described in the previous sections.

### 2.7 Degradation of Different Organic Contaminants

The capacity of the PEC/aPMS system was assessed by degrading three distinct contaminants: methylene blue (MB), tetracycline (TC), and ibuprofen (IBP). In each case, 25 mL of a 10 mg L^–1^ total organic carbon (TOC) solution (in 0.1 M Na_2_SO_4_) was introduced into the degradation cell. This concentration of contaminants is equivalent to 16.6 mg L^–1^ MB, 17 mg L^–1^ TC, and 13.9 mg L^–1^ IBP. A volume of PMS stock solution was added to achieve a final molar concentration 50 times that of the contaminant. Subsequently, simultaneous application of UV light at 365 nm and an electrochemical potential of +0.5 V vs Ag/AgCl was initiated.

The degradation progress of MB and TC was monitored using molecular absorption spectrophotometry at 664 and 356 nm, respectively. Samples were withdrawn at predetermined intervals, promptly analyzed, and returned to the reactor. IBP degradation was assessed using high-performance liquid chromatography (HPLC Ultimate 3000 SD, ThermoFisher) with a diode array detector at λ = 190 nm. In this case, 0.5 mL aliquots were withdrawn at predetermined intervals and combined with 50 μL of a 0.4 M sodium azide solution, acting as a quencher for singlet oxygen. Separation was achieved on a Restek C18 column (5 μm, 150 × 4.6 mm), and the mobile phase consisted of acetonitrile (70%) and 0.1 M acetic acid (30%) eluted in isocratic mode at a flow rate of 1 mL min^–1^. The retention time for the IBF was 3.59 min.

Moreover, the TOC (total organic carbon) concentration of the initial and final aliquots (60 min) was assessed to evaluate mineralization (TOC-L_CSH_, Shimadzu). Two samples were taken for the 60 min aliquot: one sample was analyzed without quenching, and the other was quenched with sodium azide (NaN_3_).

## 3 Results and Discussion

### 3.1 Metallography and Quantification of Substrate Phases

The stoichiometric composition of the Ti–5.5Cu alloy substrate was measured by XRF, and the values in mass percentage were 92.82% and 7.18%, which correspond to 94.49 (±0.01) and 5.51 (±0.01) in atomic percentage for Ti and Cu, respectively. The measured and expected values of the Ti and Cu contents are in excellent agreement, indicating that the casting procedures were appropriate.

**Figure 1:**
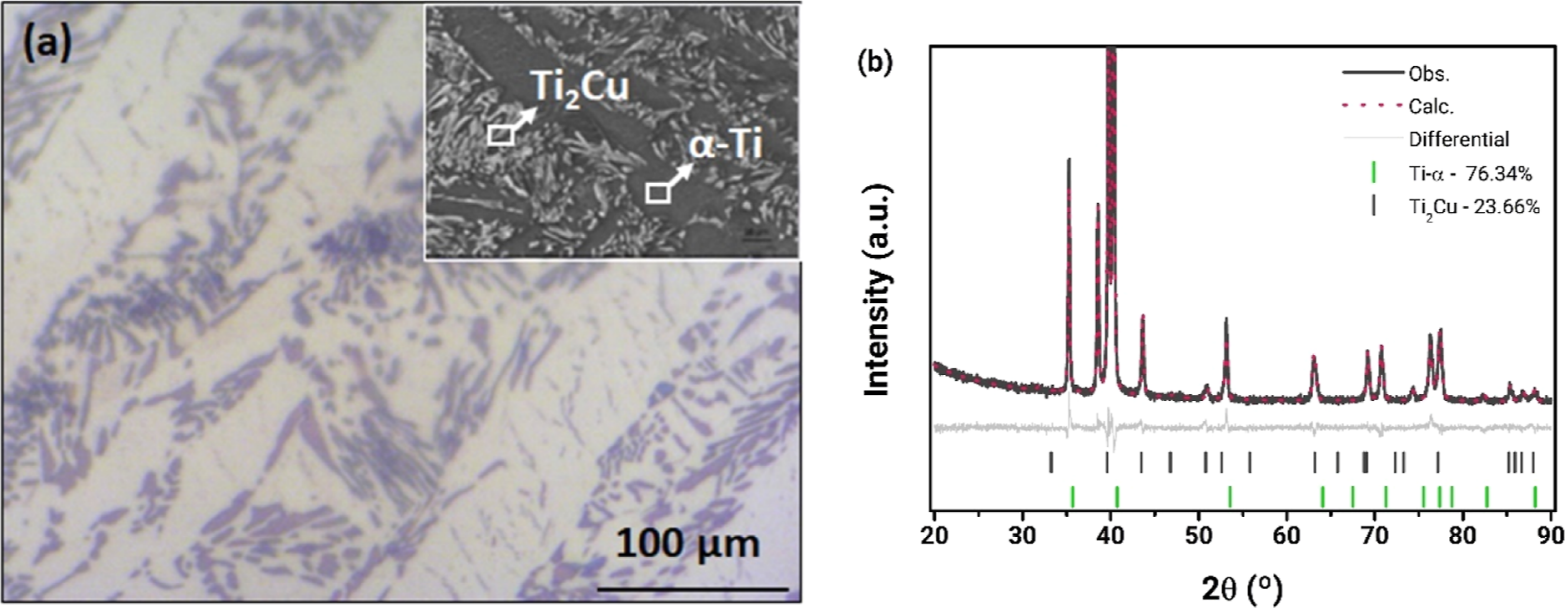
Characterization of the Ti–5.5Cu (% at.) substrate. (a) Optical microscopy image and scanning electron micrograph of the annealed substrate microstructure in detail. (b) X-ray diffractometry pattern and phase quantification obtained from Rietveld refinement.

Figure 1a shows an optical micrograph and inset scanning electron microscopy image obtained from the Ti–5.5Cu (at. %) substrate. The optical image exhibits two different fields, corresponding to a lenticular region separated by darker fields and the opposite color on the electron image. From EDX analysis, the lenticular region was associated with the primary α-Ti (1—dark phase), and Ti_2_Cu was related to the precipitate (2—white phase), as predicted by the Ti–Cu phase diagram for this composition.^23^

The X-ray diffraction measurement was carried out to corroborate the phases identified by EDX analysis for Ti–5.5Cu (at. %). The data were compared with the standard XRD patterns, and Rietveld refinement (Figure 1b) was used to determine the quantification of phases that comprise the substrate. Rietveld refinements of the XRD data showed two phases, indicating that Ti-α (*P*6_3_/*mmc*) was a majority phase of Ti–5.5Cu (at. %) (∼76.4%) with the minor phase of Ti_2_Cu (*I*4/*mmm*) (∼23.7%), in an approximate 3:1 ratio composition. The refinement parameters obtained were as follows: residual profile (*R*_p_ = 4.14), expected residual (*R*_exp_ = 4.03), weighted residual (*R*_wp_ = 5.40), and goodness of fit (*X*^2^ = 1.34). The Ti-α and Ti_2_Cu phases are equilibrium phases in the Ti–Cu system. However, only Ti-α can dissolve copper below the solubility limit in the Ti–Cu system (≤1.6 at. % Cu), forming a solid solution.^23^ All analyses carried out on the Ti–5.5Cu (at. %) substrate suggest the composition α-Ti as the predominant phase, α-Ti doped with copper and Ti_2_Cu precipitate.^24^

### 3.2 Characteristics of TNT and Ti–O–CuNT Electrodes

#### 3.2.1 Morphological and Crystallographic Characterization

**Figure 2:**
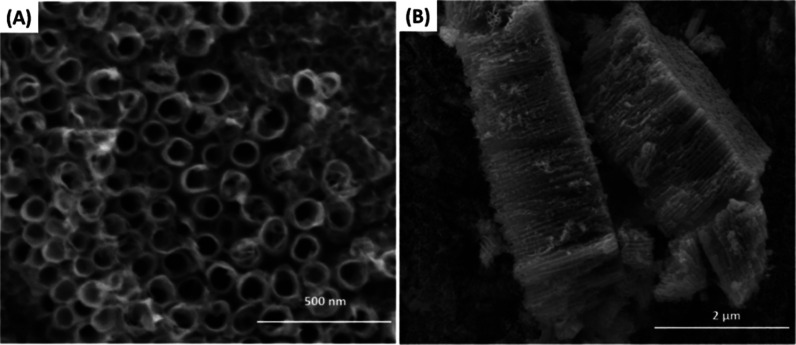
Scanning electron microscopy (SEM) images of the Ti–O–Cu nanotubular oxide film (Ti–O–CuNT) grown on a Ti–5.5Cu (at. %) alloy substrate by electrochemical anodization and annealed at 450 °C. (A) Top view showing the nanotube structure. (B) Cross-section illustrating the film thickness and nanotube architecture.

Electrochemical anodization of the Ti–5.5Cu (atom %) alloy followed by annealing at 450 °C produced well-ordered Ti–O–Cu mixed oxide nanotubes as observed by SEM (Figure 2). The uniform nanotube morphology with an inner diameter of ∼70 nm, wall thickness of 12 nm, and length of 2 μm provides a high surface area, beneficial for enhanced mass transport and light absorption in photoelectrocatalysis.

**Figure 3:**
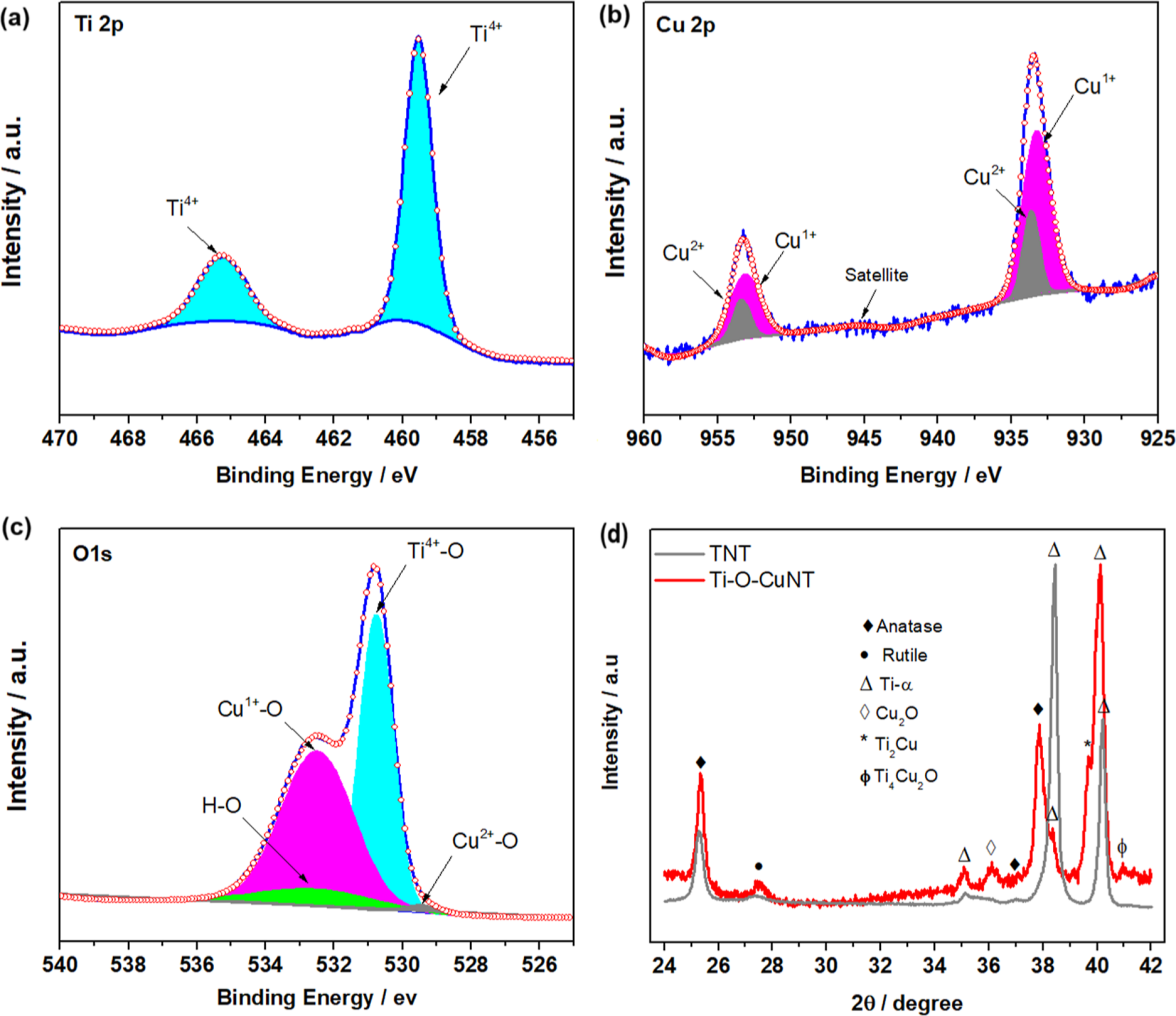
Analysis of layer composition and crystalline structures of Ti–O–CuNT annealed at 450 °C: (a–c) XPS spectrum of Ti 2p, Cu 2p, and O 1s and (d) XRD profiles of Ti–O–CuNT compared with TNT.

XPS analysis confirmed the presence and chemical states of Ti, Cu, and O on the film surface (Figure 3). The Ti, Cu, and O species were confirmed on the film surface by the peaks in the Ti 2p, Cu 2p, and O 1s binding energy regions, respectively (Figure 3a–c). For Ti 2p spectra (Figure 3a), the peaks at 459.5 eV (Ti 2p_3/2_) and 465.3 eV (Ti 2p_1/2_) correspond to the Ti^4+^ and its doublet, respectively, separated by 5.8 eV. However, these peaks shifted by 1.0 eV when compared with the reference standard,^25^ indicating the presence of impurity dopants in the TiO_2_ lattice.

The Cu 2p spectra showed signatures of Cu^1+^ and Cu^2+^ (Figure 3b), which are assigned with Cu 2p_3/2_ and Cu 2p_1/2_, respectively, derived from copper oxide. The deconvolution of this spectrum revealed that the material is composed of a mix of valences between Cu^1+^ (933.1/933.6 eV) and Cu^2+^ (953.0/953.3 eV),^25^ where the Cu^+^ species was predominant on the composition in relation to Cu^2+^. Despite the detected Cu 2p_1/2_ peak and its respective doublet Cu 2p_3/2_ from Cu^2+^, the shakeup satellite characteristic of this species was absent. The absence of shakeup satellite peaks in the Cu^2+^ spectrum may be associated with a high density of oxygen vacancies in the oxide composition.^26^

The O 1s binding energy region of Ti–O–CuNT is shown in Figure 3c, which is fitted with four peaks after deconvolution. The peaks at binding energies 530.7, 532.5, and 529.4 eV are attributed to the oxygen atoms bound to Ti^4+^, Cu^1+^, and Cu^2+^ species, respectively.^27^ Meanwhile, the peak of 532.6 eV can be ascribed to oxygen bound to H due to the interaction of the sample with the atmospheric air.^28^ Furthermore, a shift of peaks at ∼0.9 eV was observed, which may indicate a TiO_2_ doping process and/or the formation of a mixed valence oxide between Ti–O–Cu.^29,30^

From the diffraction data obtained for Ti–O–CuNT, crystalline phases of TiO_2_ (anatase and rutile), Cu_2_O, and nonstoichiometric Ti_4_Cu_2_O were obtained, with the anatase phase being the predominant. The anatase is the most photoactive and desirable TiO_2_ phase in photoelectrocatalytic applications due to its lower charge recombination rate and greater charge carrier mobility.^31^ Also, XRD data revealed only the incidence of Cu^1+^, whereas the peak of Cu^2+^ was suppressed, which may be due to its lower concentration on the composition of the film grown on Ti–5.5Cu (at. %). This result was similar to XPS data, indicating a higher concentration of Cu^1+^ than Cu^2+^.

#### 3.2.2 Photoelectrochemical Characterization

**Figure 4:**
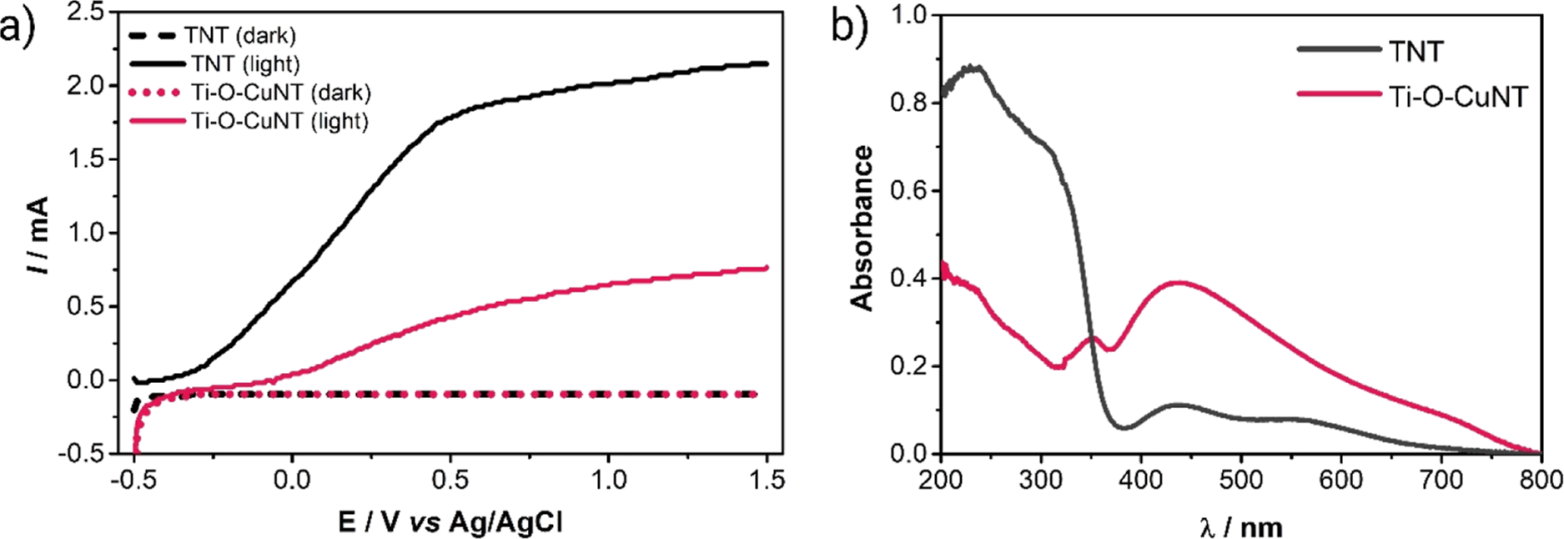
(a) Linear scan voltammograms for TNT and Ti–O–CuNT electrodes under irradiation and in the dark. Conditions: 0.1 M Na_2_SO_4_ electrolyte; scan rate of 20 mV s^–1^; radiation from a 10 W UV 365 nm LED. (b) Absorption spectra obtained by diffuse reflectance spectroscopy for UV and visible regions for TNT and Ti–O–CuNT samples.

Linear scanning voltammograms of the TNT and Ti–O–CuNT electrodes, in the dark and under irradiation (10 W UV 365 nm LED) (photocurrent), are shown in Figure 4a. At both electrodes, an approximately zero anodic current is displayed without irradiation, as expected for an *n*-type semiconductor. However, when the TNT electrode is irradiated, the current increases linearly from −0.25 to +0.5 V (vs Ag/AgCl), reaching about 1.8 mA and becoming approximately constant. The Ti–O–CuNT electrode showed a significantly lower photocurrent than the electrode without copper doping. This behavior can be explained by the absorptivity of the materials in the ultraviolet region, as shown in Figure 4b (obtained by diffuse reflectance spectroscopy). The absorption spectrum of the TNT sample clearly shows that its absorbance is much higher than that of Ti–O–CuNT in the region from 200 to 350 nm.

### 3.3 Simultaneous Photoelectrocatalytic Oxidation and PMS Activation by the Ti–O–CuNT Electrode

#### 3.3.1 Experimental Design for Optimization of PEC MB Degradation Conditions

**Figure 5:**
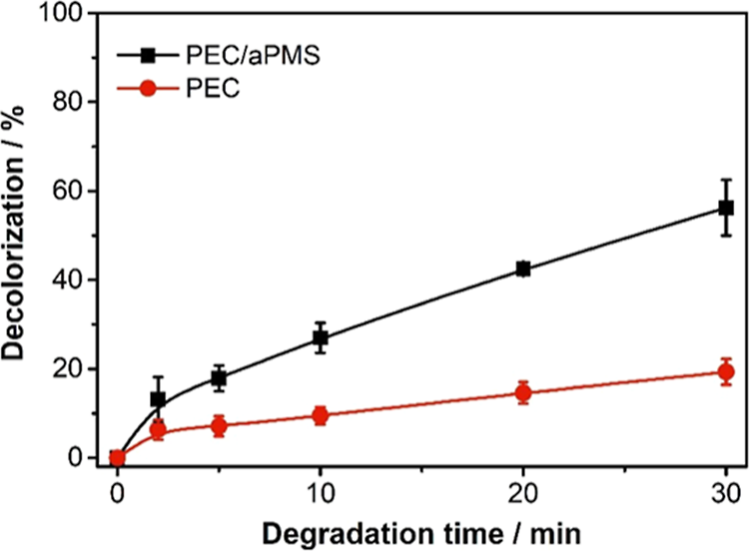
Decolorization of a 5 mg L^–1^ MB solution by photoelectrocatalysis (PEC) and photoelectrocatalysis with activated PMS (PEC/aPMS) using a Ti–O–CuNT photoanode. Conditions: 25 mL of MB 5 mg L^–1^ in Na_2_SO_4_ 0.1 M; [PMS] = 0.468 mM; pH 6.5; 1.5 V vs Ag/AgCl potential; constant agitation of 1000 rpm; irradiation by a 10 W UV LED at 365 nm; spectrophotometric analysis at 664 nm.

Initially, it was found that the Ti–O–Cu mixed oxide electrode can activate PMS in a photoelectrocatalytic system (Figure 5). In the absence of PMS, there was a photoelectrocatalytic decolorization of 19% in 30 min, while in the presence of PMS, it reached 56%. Then, the degradation conditions were optimized through a 2^3^ FFED using the independent variables, and their ranges are shown in Table 1. The pH range (i.e., from 4.00 to 8.00) was chosen because it is closer to neutral pH and fits most wastewater available for treatment. In addition, the SO_4_^•–^ radical has a wide operational pH range, so it is possible to use the AOPs in a range of 3.0 to 9.0.^32^ The potential range (0.5 to 1.5 V vs Ag/AgCl) refers to the lowest potential capable of generating maximum photocurrent values (see Figure 4a), while the potential of 1.5 V is the threshold value, above which the stability of the mixed oxide is affected. The concentration of PMS, the reactive species precursor, was selected within a range (0.156 to 0.780 mM) commonly used in related studies.^33^ These concentrations are equivalent to 10, 30, and 50 times the molar concentration of the contaminant (MB dye).

Using the least-squares method, a first-order polynomial model (eq 2) was constructed from the data of runs 1–19. The resulting model (eq 3) for decolorization efficiency is
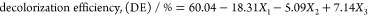
3

**Figure 6:**
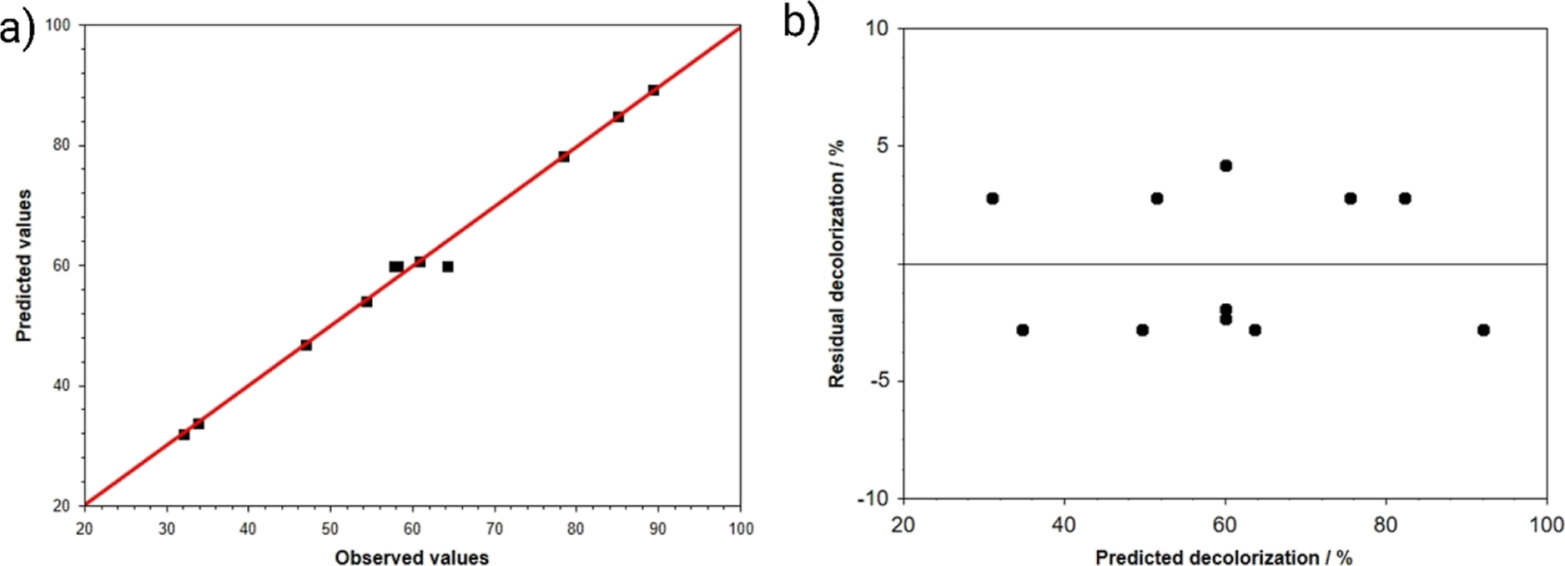
(a) Predicted–observed and (b) residual–predicted plots determined for the decolorization of a 5 mg L^–1^ of MB solution in 0.10 mol L^–1^ Na_2_SO_4_ with a Ti–O–CuNT anode irradiated by a 10 W UV LED at 365 nm in the presence of PMS (PEC/aPMS system).

Only the statistically significant terms (at a 95% confidence level) were included in eq 3, where *X*_1_, *X*_2_, and *X*_3_ represent the coded variables for pH, applied potential, and PMS concentration, respectively. Polynomial eq 3 was statistically evaluated using the analysis of variance (ANOVA) comparing the variation sources with the *F*-test (i.e., the Fisher distribution), which allows finding the polynomial with the best fit to the experimental data.^34^Figure 6a depicts a good correlation between the predicted and observed values with an *R*^2^ value equal to 0.962, whereas Figure 6b confirms the absence of systematic errors attributed to the random distribution of the residuals around the zero deviation. Therefore, these statistical results indicate that the model is statistically significant (at a 95% confidence level) (see also Table S1).^18^

Analyzing polynomial eq 3, it is observed that the pH value, applied potential, and PMS concentration significantly influenced the decolorization efficiency. Equation 3 also shows that the decrease in pH causes a higher decolorization efficiency, indicated by the highly negative value of the coded variable *X*_1_, which is the most significant variable. This effect is attributed to the electrostatic interaction between the surface charges of the Ti–O–CuNT electrode and the ionic form of PMS (HSO_5_^–^). The point of zero charge of TiO_2_ is known to be found at pH 5.9.^35^ Therefore, at pH 4.0, the electrode surface is positively charged, and PMS exists as an anion (HSO_5_^–^/SO_5_^2–^ with p*K*_a_ = 9.4).^32^ Consequently, more PMS is adsorbed on the electrode surface, increasing the formation of the PMS–Ti–O–Cu complex and facilitating the PMS catalysis cycle with the copper catalytic sites. Regarding the concentration of PMS, there is a positive effect on the response when its concentration is increased as this implies a greater generation of reactive species that act directly on the degradation of the MB dye.

**Figure 7:**
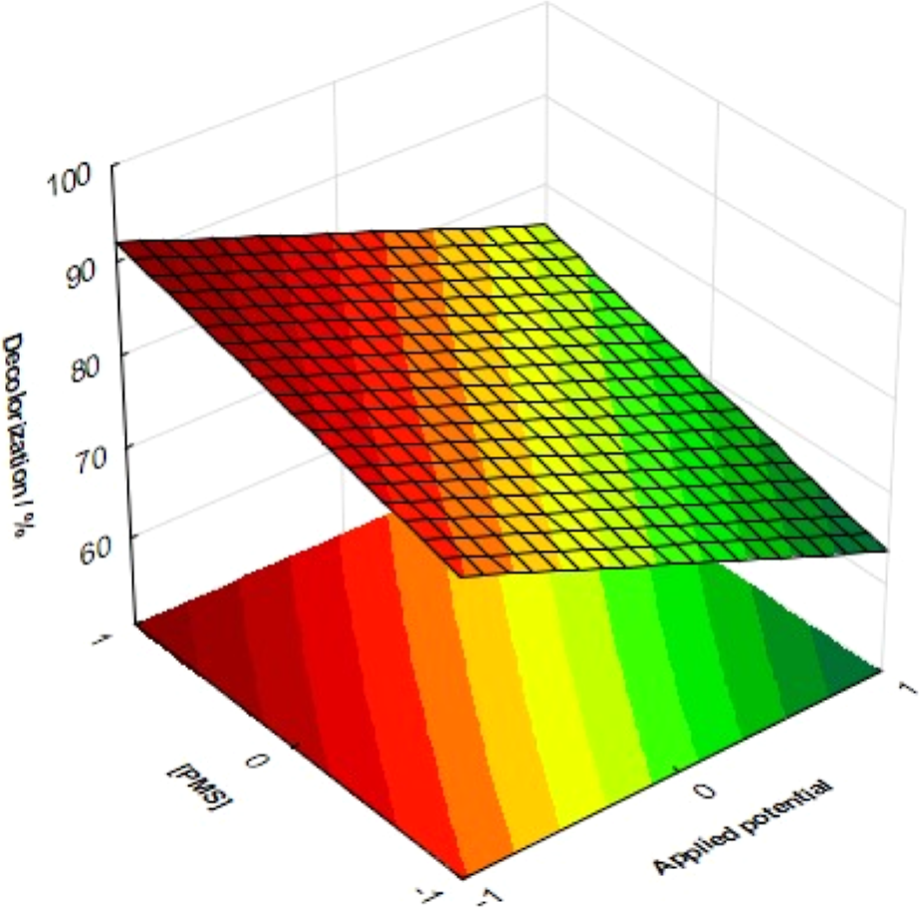
Response surface generated from a full factorial design expressing the decolorization efficiency as a function of PMS concentration and applied potential after 30 min of PEC/aPMS treatment. Conditions: 25 mL of 5.0 mg L^–1^ of MB solution in 0.1 M Na_2_SO_4_ with a Ti–O–CuNT anode, potential of 0.5 V vs Ag/AgCl, irradiated by a 10 W UV LED at 365 nm. Independent variable pH solution was set at coded level −1 (i.e., at pH 4.0).

Therefore, considering the negative effect of pH on the decolorization, added to the fact that the interactions between the investigated variables were not statistically significant, only the response surface for decolorization as a function of applied potential and PMS concentration was investigated, setting the coded variable *X*_1_ at the lowest level (i.e., at pH 4.0), as previously justified. Figure 7 shows the response surface related to DE (in %) as a function of the applied potential and PMS concentration. As can be seen, the increase in PMS concentration causes a higher DE for all applied potential values, with a maximum decolorization efficiency of about 94% after 30 min of PEC/aPMS treatment of MB solution for a higher PMS concentration (which is 50 times the molar concentration of the dye) and lower applied potential. Furthermore, eq 3 and the response surface show that DE increased linearly with the increase in the PMS concentration and the decrease in the applied potential in the investigated intervals.

As is already known, irradiating the TiO_2_ and other semiconductors with photons of appropriate energy generates electron–hole pairs. Using a photoelectrocatalytic system, with the application of a potential difference, accelerates the transfer of electrons from the semiconductor to the counter electrode, reducing the recombination of charges in relation to a purely photocatalytic (PC) system.^17^ A lower potential level (0.5 V vs Ag/AgCl) was observed to contribute positively to more significant MB degradation. As observed in the photocurrent curve (Figure 4a), this potential can produce practically the maximum photocurrent and favors the activation of the PMS. This behavior can be attributed to the fact that more positive potentials can have a negative effect on the copper oxidation state cycle responsible for PMS catalysis. For PMS conversion, the catalyst (copper centers) must transfer an electron to the PMS that is adsorbed on the catalyst surface. Thus, with this electronic transfer, the oxidation of Cu(I) centers to Cu(II) or Cu(III) occurs. Photogenerated electrons may be able to reduce the Cu(II) or Cu(III) centers again to Cu(I) for the renewal of the catalysis cycle. Thus, higher potentials, such as 1.5 V, may disfavor this reduction process due to the high positive potential and the depletion of photogenerated electrons from the photoanode. This behavior corroborates what is observed in eq 3 (see the negative coefficient in the variable *X*_2_ for the applied potential) and the response surface generated from this polynomial equation.

These findings identify the optimal conditions for the independent variables—pH, applied potential, and PMS concentration—within the studied range, determined as pH 4.0, an applied potential of 0.5 V vs Ag/AgCl, and a PMS concentration of 0.780 mmol L^–1^ (equivalent to 50 times the MB concentration) for effective PEC/aPMS treatment of the MB solution.

#### 3.3.2 Comparison between Various Treatment Combinations

**Figure 8:**
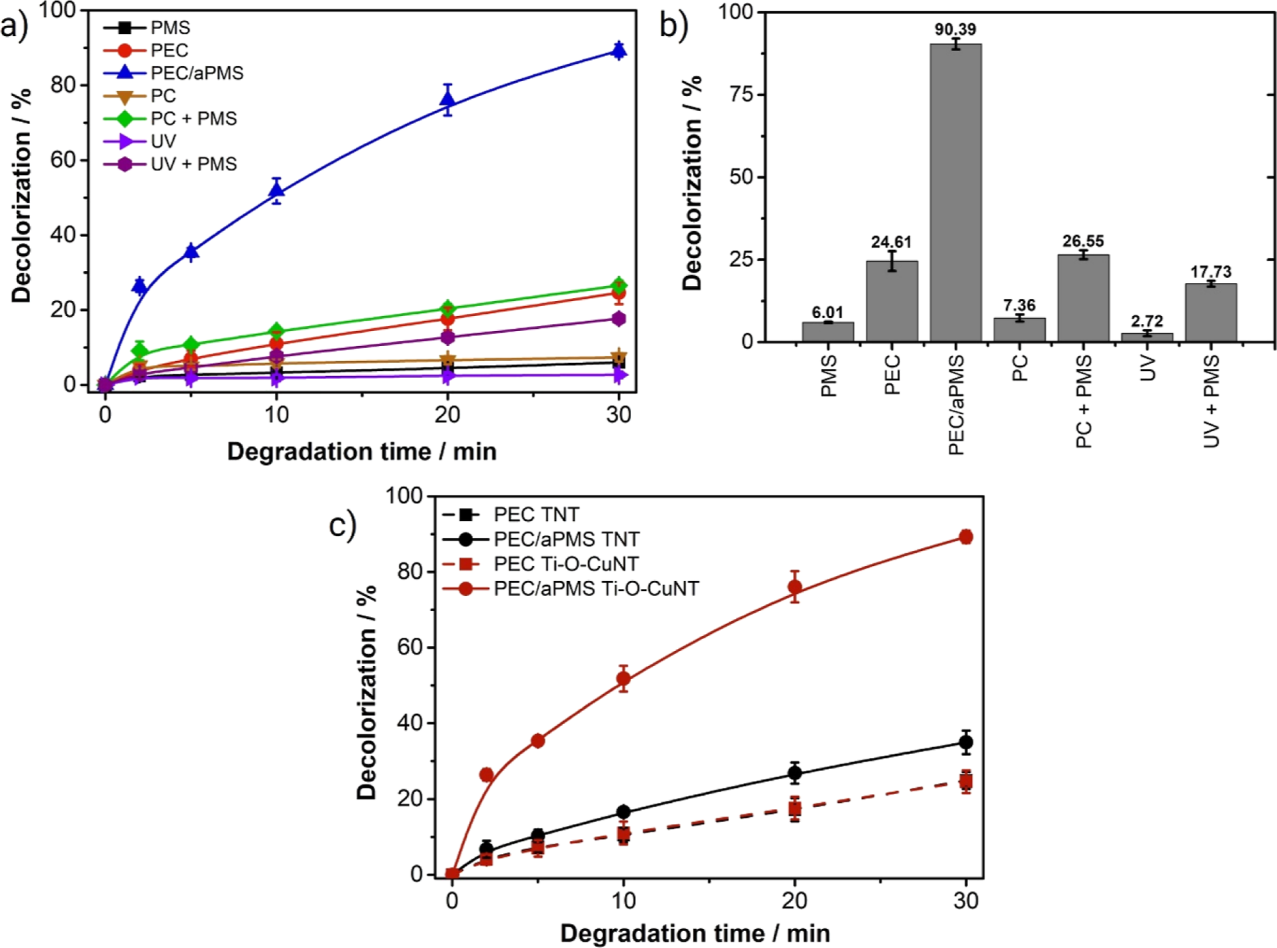
(a) Comparison of decolorization of MB dye solution by different systems. Common conditions: 25 mL of 5 mg L^–1^ MB in 0.1 M Na_2_SO_4_ at pH 4, under constant stirring. The independent conditions were as follows: Ti–O–CuNT electrode, [PMS] = 0.780 mM, potential of 0.5 V vs Ag/AgCl, 10 W UV LED irradiation at 365 nm; (b) % of decolorization in 30 min of treatment by the different evaluated systems. (c) Comparison of decolorization of MB dye solution by PEC (■) and PEC/aPMS (●) with Ti–O–CuNT (red) and TNT (black) electrodes.

After the optimization of some conditions of PEC/aPMS treatment (pH 4, potential 0.5 V vs Ag/AgCl, and [PMS] = 0.780 mM), several different combinations and isolated systems were evaluated for comparison purposes (Figure 8a,b). For each electrode use, a “regeneration” step was performed by cyclic voltammetry in a 0.1 M Na_2_SO_4_ solution (50 cycles) under UV irradiation, ensuring good repeatability. No decrease in the material efficiency was observed.

Direct oxidation by PMS resulted in only 6% decolorization of the MB solution at 30 min due to the direct redox reaction (*E*^0^ = 1.85 V).^36^ The photolysis of the dye is negligible, obtaining a decolorization of only 3%, showing that the dye is relatively stable under 365 nm UV irradiation. Combining irradiation with PMS (photolysis + PMS) resulted in a decolorization of 17%, indicating that UV light allowed an increase of 9%. This may be attributed to the activation of PMS by radiation (HSO_5_^–^ + *h*ν → HO^•^ + SO_4_^•–^), which generated additional reactive species, albeit at a negligible scale.^37^

The photocatalytic (PC) decolorization using Ti–O–CuNT and light was only 7%, but it improved to 27% with the addition of PMS due to generating additional reactive species. The photoelectrocatalysis (PEC) treatment showed a decolorization of 25% because of the more effective charge separation compared to PC. However, adding PMS to the PEC system resulted in a substantial improvement of 90% in decolorization, revealing a synergistic effect. This significant increase in decolorization efficiency is attributed to the formation of additional reactive species, including radicals (^•^OH, SO_4_^•–^, and/or O_2_^•–^), PMS activated at the surface, or singlet oxygen (^1^O_2_). These mechanistic details will be discussed later.

Finally, it was verified whether the copper in the mixed Ti–O–CuNT electrode was responsible for activating the PMS. For this, the PEC/aPMS and PEC systems were investigated using the TNT and Ti–O–CuNT electrodes (Figure 8c). It was found that PEC treatment resulted in 25% decolorization with both electrodes. In PEC/aPMS with the copper-free electrode (TNT), a decolorization of 35% was obtained. Thus, adding PMS resulted in a 10% increase, which can be attributed to the activation of PMS by light but not necessarily due to the conversion of PMS by TiO_2_-based PEC. It is only in the PEC/aPMS system with the Ti–O–CuNT electrode that a significant efficiency improvement is observed (to 90.4%), proving that the mixed oxide (Cu_2_Ti_4_O and Cu_2_O, Figure 3) acts directly in PMS catalysis. These results also confirm no activation of PMS by the stainless-steel cathode, as reported by other works using platinum cathodes.^11^

### 3.4 Mechanism of Generation of Reactive Species

Using scavengers of free radicals and other reactive species is essential for studying their actions in advanced oxidation systems.^38^ Methanol (MeOH) interacts similarly with sulfate radicals (SO_4_^•–^) and hydroxyl radicals (^•^OH), having rate constants of 9.7 × 10^8^ M^–1^ s^–1^ and 1.0 × 10^7^ M^–1^ s^–1^, respectively; hence, its use suggests the involvement of radical reactions when MB degradation is inhibited.^21^ Conversely, *tert*-butyl alcohol (TBA) predominantly scavenges ^•^OH due to its significantly higher rate constant with ^•^OH (3.8–7.6 × 10^8^ M^–1^ s^–1^) compared to SO_4_^•–^ (4.0–9.1 × 10^5^ M^–1^ s^–1^).^21^ Sodium azide (SA) serves as a quencher of singlet oxygen (^1^O_2_) as it can promote the nonradiative decay back to its ground state.^22^

**Figure 9:**
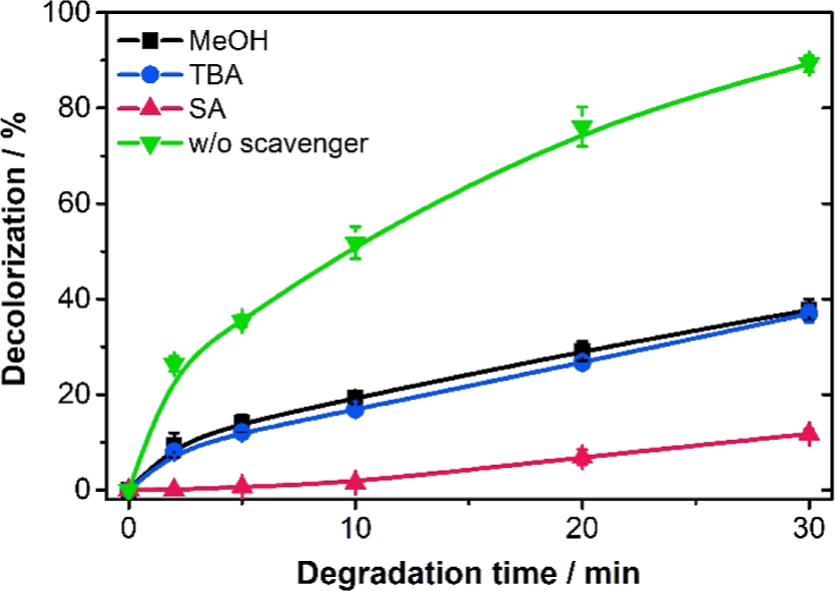
Influence of scavengers on the decolorization of MB dye solution by the PEC/aPMS system using sodium azide (SA), methyl alcohol (MeOH), and *tert*-butyl alcohol (TBA) in the proportion of 50 × [PMS] = 0.4 M. Conditions: 25 mL MB 5 mg L^–1^ in Na_2_SO_4_ 0.1 M; 0.5 V; [PMS] of 0.780 mM; pH 4; constant agitation; irradiation by a 10 W UV LED at 365 nm.

As shown in Figure 9, under optimized conditions, the PEC/aPMS system achieves a 90.4% degradation efficiency after 30 min without any scavengers. However, decolorization decreased to 37.7%, 37.1%, and 11.7% after adding MeOH, TBA, and SA, respectively. Thus, the main route of formation of reactive species via PMS activation by the Ti–O–CuNT electrode can be ascribed to the formation of the ^1^O_2_. In addition, there is a contribution of photoelectrocatalytically generated ^•^OH, but no SO_4_^•–^ was formed by PMS activation.

Considering pseudo-first-order kinetics for all curves is shown in Figure S1, the rate constants, *k*, were calculated and are shown in Table 2. From these data, the contributions of the radical and nonradical pathways to the MB decolorization by PEC/aPMS were calculated using eqs 4 and 5(9)
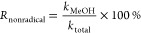
4
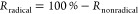
5where *R*_radical_ and *R*_nonradical_ represent the estimated contribution rates calculated for the radical and nonradical mechanisms, respectively, *k*_MeOH_ is the rate constant in the presence of the scavenger MeOH, and *k*_total_ refers to the constant in the absence of scavengers.

**Table 2:** Rate Constants (*k*) Obtained by ln[abs/abs_0_] vs Time for the Degradation of 25 mL of 5 mg L^–1^ MB in Na_2_SO_4_ 0.1 M Using PEC/aPMS in the Absence and Presence of Different Scavengers

quencher	captured species	*k* (min^–1^)	*R*^2^	contribution (%)
		0.0755	0.994	
MeOH	SO_4_^•–^ e HO^•^	0.0139	0.971	18.4
TBA	HO^•^	0.0136	0.979	18.0
sodium azide	^1^O_2_	0.0039	0.939	81.6

As the contribution percentages of MeOH and TBA scavengers were very close (about 18%), there were no captured SO_4_^•–^ radicals but only ^•^OH (generated by photoelectrocatalysis on TiO_2_). Then, the contribution of the nonradical species (^1^O_2_) was 81.6%. Therefore, the most significant contribution of decolorization is due to the nonradical pathway. However, at this moment, it is impossible to state that other species are not being generated, such as the surface-activated PMS route.^39,40^

Activation of peroxymonosulfate (PMS) through both radical and nonradical pathways results in significant differences. The nonradical oxidation reactions have a significant advantage as they are not impacted by pH, are resistant to inorganic ions and organic matter found in wastewater, reduce the formation of toxic byproducts (such as chloride and bromide), and increase the efficiency of PMS consumption.^33^

This evidence suggests a possible PMS activation mechanism by copper in the PEC/aPMS system. In the ^1^O_2_ activation pathway, SO_5_^•^/O_2_^•–^ radicals are generally considered precursor intermediates. Moreover, these species can be produced by copper oxide. X-ray photoelectron spectroscopy (XPS) of the Ti–O–CuNT sample showed that the copper is mainly in the Cu(I) oxidation state and, to a lesser extent, Cu(II). The first step of PMS activation (HSO_5_^–^) involves the formation of a high-valence oxidation state, Cu(III) (eq 6).^40,41^ Then, Cu(III) (in the form of the complex [≡Cu(III)–O–H]) reacts with PMS (HO–O–SO_3_^–^), forming the metastable complex [≡Cu(III)–O–O–SO_3_] (eq 7). Then, there is an electron transfer from the PMS (a ligand) to the metal, producing O_2_^•–^ (eq 8).^40^ Subsequently, O_2_^•–^ is oxidized by Cu(III) sites, generating ^1^O_2_ (eqs 9 and 10), which is thermodynamically possible (*E*^0^_Cu(III)/Cu(II)_ = 2.3 V vs  0.79 V).^41^ Another possible reaction to generate ^1^O_2_ occurs by recombining two O_2_^•–^ (eq 11).^40^ Finally, ^1^O_2_ acts directly on the MB decolorization.
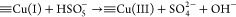
6
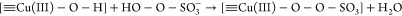
7
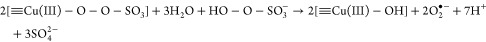
8
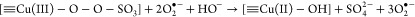
9
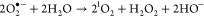
10
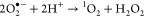
11

Additional ^1^O_2_ production route is from SO_5_^•–^. This can happen through direct one-electron oxidation of PMS by both Cu(III) and Cu(II) (eqs 12 and 13). Thus, the recombination of two SO_5_^•–^ radicals occurs, forming ^1^O_2_ (eq 14). Furthermore, the combination of SO_5_^•–^ and SO_4_^•–^ radicals can also occur to generate ^1^O_2_ (eq 15).^40^
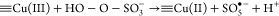
12
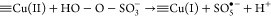
13
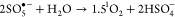
14
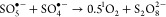
15

**Figure 10:**
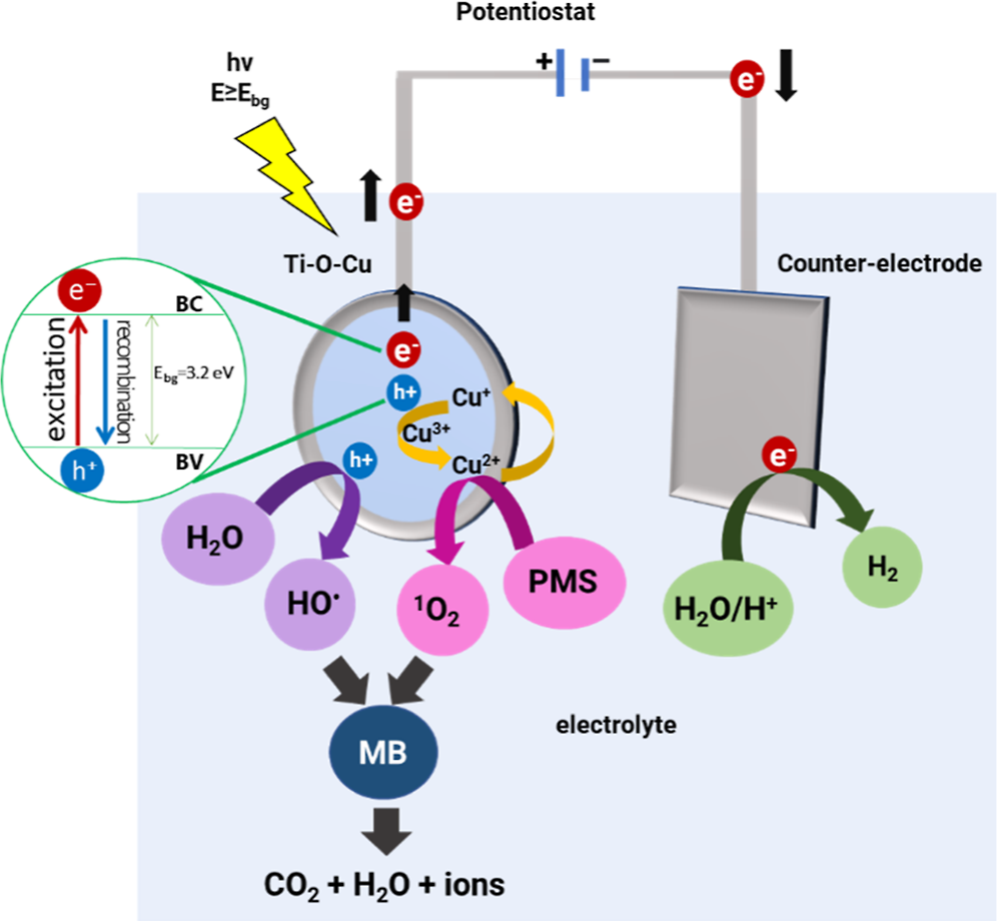
Proposed mechanism for generating reactive species in the photoelectrocatalytic system with the Ti–O–CuNT electrode in the presence of PMS (PEC/aPMS) during the degradation of methylene blue (MB) dye under irradiation. The mechanism was based on the results of experiments with different scavengers, and the detailed chemical reactions are presented in eqs 6–15.

Although it is not entirely possible to rule out the activation of PMS through radical pathways due to the occurrence of multiple simultaneous reactions, the evidence obtained from experiments using scavengers suggests that the radical component of the PEC/aPMS system primarily stems from the photoelectrocatalytic process. In this process, the photogenerated holes oxidize water molecules and create radicals ^•^OH. Therefore, Figure 10 illustrates the proposed mechanism for generating reactive species in the PEC/aPMS system.

### 3.5 Degradation of the Drugs Ibuprofen and Tetracycline for Proof of Concept

**Figure 11:**
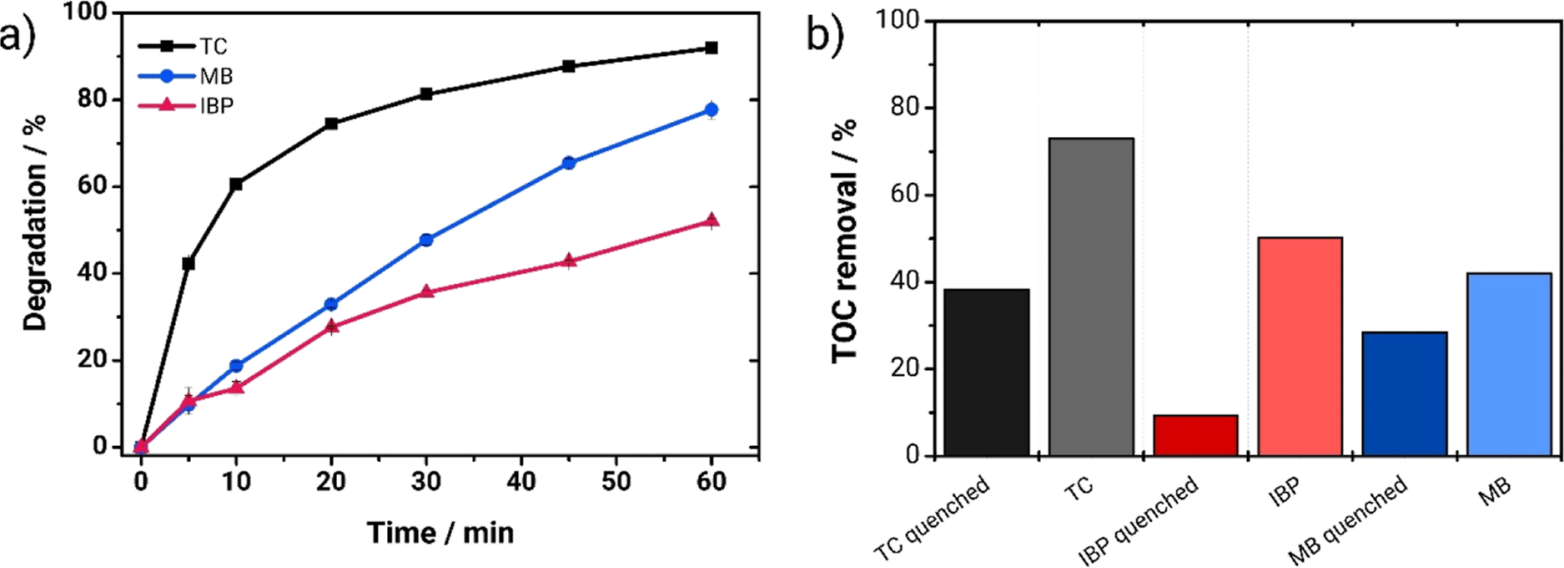
(a) Degradation (%) vs treatment time and (b) removal of total organic carbon (TOC) at 60 min for the treatment of different contaminants by the PEC/aPMS system: tetracycline (TC), methylene blue (MB), and ibuprofen (IBP), all 10 mg L^–1^ in total organic carbon in 0.1 M Na_2_SO_4_. Conditions: [PMS] = 50 × [contaminant]; pH 4; constant agitation; irradiation by a 10 W UV LED at 365 nm. TOC samples were also quenched using sodium azide to stop reactions with reactive species.

To demonstrate the broad applicability of the PEC/aPMS system, we evaluated its effectiveness against tetracycline (TC) and ibuprofen (IBP) alongside methylene blue (MB) (all 10 mg L^–1^ total organic carbon (TOC) solution in 0.1 M Na_2_SO_4_). As shown in Figure 11a, the system achieved significant degradation for all contaminants within 60 min, reaching 78, 52, and 92% for MB, IBP, and TC, respectively. These results highlight the system’s ability to degrade structurally diverse organic pollutants.

Finally, we tested that the PEC/aPMS system can activate PMS and significantly increase the photoelectrocatalytic system’s efficiency with two other model contaminants: tetracycline and ibuprofen. Figure 11a illustrates the percentage degradation over a 60 min treatment period, while Figure 11b depicts the mineralization at the end of this duration. The photoelectrocatalytic system can degrade all the contaminants, which have very different chemical structures, reaching 78, 52, and 92% degradation for MB, IBP, and TC, respectively. On the other hand, TOC measurements showed an interesting behavior: when we quenched the 60 min sample with sodium azide, the mineralization was 28, 9, and 39% for MB, IBP, and TC, respectively. However, another sample that was not quenched was also measured, reaching 42, 50, and 73%, respectively. This result further confirms the nonradical mechanism, which has slower kinetics. This result confirms that singlet oxygen is generated since the surface-activated PMS route cannot occur without the catalyst material. Singlet oxygen is less efficient than radicals (such as sulfate/hydroxyl) in completely oxidizing contaminants. While less efficient than radicals in complete oxidation, singlet oxygen offers advantages like resistance to common wastewater contaminants and reduced formation of harmful byproducts.^40^

To further evaluate the performance of the Ti–O–CuNT electrode in the PEC/aPMS system, we conducted a comparison to recently published studies using similar materials (Table 3). Our system utilized contaminant concentrations that were even higher than those in other comparable studies, along with a less powerful, cost-effective light source that is more energy-efficient. Additionally, we maintained a compatible concentration of PMS, achieving excellent removal percentages for the contaminants. These results underscore the robustness of our PEC/aPMS system compared to other advanced photoelectrocatalytic technologies. Notably, our system demonstrated high stability and reusability as a single electrode was used for all experiments. In the event of electrode deactivation, the oxide layer can be easily removed, allowing for reanodization to regenerate a new catalytic oxide without complex procedures, excessive reagent costs, or waste generation. The table highlights the versatility of our system, particularly in its ability to effectively degrade structurally diverse pollutants, positioning it as a competitive option for wastewater treatment applications.

**Table 3:** Comparative Analysis of Degradation Efficiencies of Various Contaminants by Photoelectrocatalysis-Assisted PMS Activation by Photoanodes

photoanode material	contaminant	[PMS]	light source	potential	degradation %	reference
Ti–O–Cu nanotubular mixed oxide grown on a TiCu alloy	methylene blue, MB (16.6 mg L^–1^)	2.6 mM	10 W UV LED (365 nm)	0.5 V vs Ag/AgCl	78% (MB)	this work
	ibuprofen, IBP (13.9 mg L^–1^)	3.0 mM			52% (IBP)	
	tetracycline, TC (17 mg L^–1^)	1.7 mM			92% (TC) (60 min)	
MoS_2_@C/CC	norfloxacin (2 mg L^–1^)	40 ppm (0.13 mM)	350 W xenon lamp with a 420 nm cutoff filter	1.5 V	100% (30 min)	(15)
g-C_3_N_4_/TiO_2_ nanotube arrays	tetracycline (10 mg L^–1^)	2 mM	500 W xenon lamp	2.0 V	95.69% (60 min)	(42)
BiVO_4_	bisphenol A (10 mg L^–1^)	5 mM	300 W Xe lamp with a 420 nm cutoff filter	0.25 V vs SCE	100% (120 min)	(43)
FTO-Bi_2_WO_6_	sulfamethoxazole, SMX (5 mg L^–1^)	3 mM	300 W xenon lamp with a AM1.5G filter	1.0 V vs Ag/AgCl	98% (SMX)	(44)
	tetracycline, TC (5 mg L^–1^)				79% (TC)	
	diclofenac chloride, DC (5 mg L^–1^)				83% (DC) (90 min)	
TiO_2_/WO_3_	17α-ethinyl estradiol (1 mg L^–1^)	10 mg L^–1^	100 W xenon ozone-free solar simulator	1.0 V vs Ag/AgCl	88.8% (60 min)	(45)
Co-BiVO_4_	bisphenol A (20 mg L^–1^)	2 mM	300 W Xe-lamp (100 mW cm^–2^)	1.2 V	99.16% (60 min)	(16)
CoFe_2_O_4_–BiVO_4_	tetracycline (20 mg L^–1^)	0.5 mM	300 W Xe lamp with a 420 nm cutoff filter	0.6 V vs RHE	89.1% (60 min)	(46)
Sn-doped α-Fe_2_O_3_	2-(4-isobutylphenyl)propanoic acid (10 mg L^–1^)	5 mM	visible LED 400 nm, 6 mW cm^–2^	1.5 V vs RHE	99% (210 min)	(47)

## 4 Conclusions

In an unprecedented way, we showed that it is possible to activate PMS by the photoanode of a photoelectrochemical system consisting of a Ti–O–Cu mixed oxide nanotube. Through a 2^3^ experimental design and surface response, the conditions of the combined PEC/aPMS system showed superior activity at pH 4, an applied potential of 0.5 V vs Ag/AgCl, and a PMS concentration of 0.780 mM (50× the molar concentration of the contaminant). In only 30 min of treatment, 90.4% MB decolorization (5 mg L^–1^) was achieved by the PEC/aPMS system. Additionally, it was demonstrated that PMS activation occurs mainly through nonradical pathways, likely through the formation of singlet oxygen, ^1^O_2_. Due to its preparation method (anodization of a Ti–Cu alloy), the catalyst (copper) is firmly bonded to the TiO_2_ crystal structure, and the electrodes showed excellent stability. There is still a need to assess the toxicity of the treated sample, the efficiency in the presence of real contaminated matrices, and the scale-up of the system, which will be evaluated in the future stages. These results are an essential step in the search for more efficient advanced oxidative processes, showing a new approach for applying high-stability mixed oxide electrodes prepared from metal alloys.

## Supporting Information Available

Analysis of variance (ANOVA) of data experimental design and curves of ln[abs/abs_0_] vs time (kinetics) (PDF)
