# Supplementary material for: Ti–O–Cu Nanotubular Mixed Oxide Grown on a TiCu Alloy as an Efficient Material for Simultaneous Photoelectrocatalytic Oxidation and PMS Activation for Pollutant Degradation
Source: ACS Omega. 2024 Nov 13;9(47):47052–64. doi: 10.1021/acsomega.4c07301 (PMC11603227; doi:10.1021/acsomega.4c07301)
Supplement: Supplementary file 1 — ao4c07301_si_001.pdf [file ao4c07301_si_001.pdf]

Supporting Information

Ti-O-Cu nanotubular mixed oxide grown on TiCu alloy as an efficient material for simultaneous photoelectrocatalytic oxidation and PMS activation for pollutant degradation

*Carolyne Izaira Prates Crivelli[a], Juliana de Almeida[b], Cleber Antonio Lindino[a], Lucio Cesar de Almeida[c], Christiane de Arruda Rodrigues[b], Guilherme Garcia Bessegato[*d]*

[a]Universidade Estadual do Oeste do Paraná (UNIOESTE), Rua da Faculdade 645, 85903-000 Toledo, PR, Brazil.

[b]Universidade Federal de São Paulo (UNIFESP), Rua São Nicolau 210, 09913-030 Diadema, SP, Brazil.

[c]Universidade Estadual de Londrina (UEL), Laboratory of Environmental Electrochemistry – LabEA, Department of Chemistry, Rodovia Celso Garcia Cid, PR 445 km 380, 86057-970, Londrina, PR, Brazil.

[d]Universidade Tecnológica Federal do Paraná (UTFPR), Dois Vizinhos Campus, Estrada para Boa Esperança, km 04, 85660-000 Dois Vizinhos, PR, Brazil.

*E-mail: bessegato@utfpr.edu.br

*Phone number: +554635368209

Experimental design for optimization of PEC MB degradation conditions

Table S1 shows the values identified for analysis of variance (ANOVA). It also shows whether there is a distinction between the levels (higher or lower) of the factors, that is, whether the levels influence the response. If this influence exists, then the factor is considered significant. This can be verified by observing the *p*-value in Table S1: the factor is significant for *p*-values smaller than alpha (0.05). In this scenario, the pH, potential, PMS concentration, and the interaction between pH and PMS concentration are significant.

Table S1. Analysis of variance (ANOVA) of data obtained for the optimization of the PEC/aPMS system applied in the degradation of a 5 mg L^{-1} solution of MB dye by a 2^3 experimental design with a central point

	**sum of squares (SS)**	**Degrees of freedom (υ)**	**mean square (MS)**	**F-value**	**p-value**
**Curvatr.**	0.001	1	0.001	0.0000	0.995329
**(1)pH**	5366.293	1	5366.293	274.1542	0.000000
**(2)Potential**	413.212	1	413.212	21.1103	0.000772
**(3)[PMS]**	815.542	1	815.542	41.6646	0.000047
**1 by 2**	216.948	1	216.948	11.0835	0.006721
**1 by 3**	45.839	1	45.839	2.3419	0.154176
**2 by 3**	4.218	1	4.218	0.2155	0.651531
**Error**	215.314	11	19.574		
**Total SS**	7077.368	18			
**R^2**	0.96958				

Kinetic curves to calculate rate constants (*k*) of Table 3

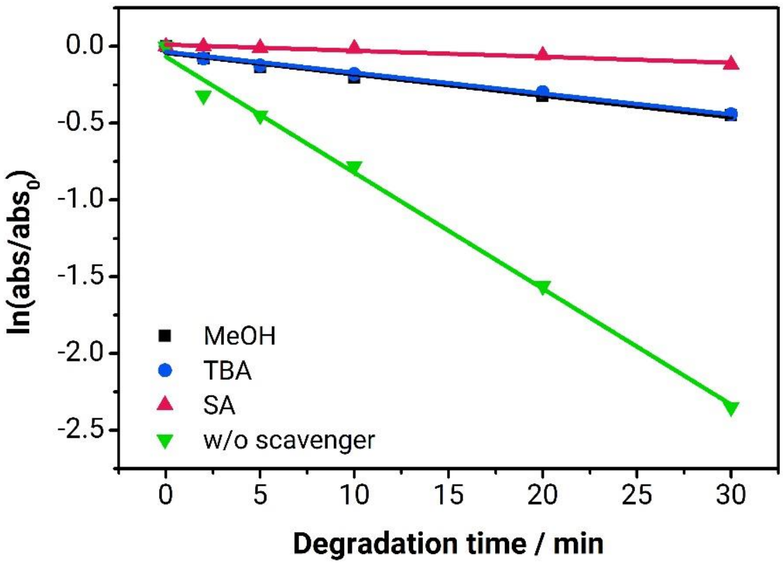

Figure S1. Curves of ln[abs/abs0] *vs*. time, for the decolorization of 25 mL of a 5 mg L^{-1} MB in 0.1 M Na_2SO_4 by the PEC/aPMS system using Ti-O-CuNT electrode, with and without the addition of scavengers (MeOH and sodium azide, SA). Conditions: the photoanode was biased at 0.5 V *vs* Ag/AgCl and irradiated by a 10 W UV 365 nm LED; [PMS] = 0.780 mM; pH 4. The concentration of the scavengers was 50× [PMS] (0.4 M).
